# Welfare Under Transient Pressure: Rethinking Behavioral Assessments and Stress Mitigation for Shelter Dogs

**DOI:** 10.3390/ani16182820

**Published:** 2026-09-08

**Authors:** Anna Breithaupt, Jacquelyn Jacobs

**Affiliations:** Department of Animal Science, Michigan State University, East Lansing, MI 48824, USA; breitha8@msu.edu

**Keywords:** shelter dogs, stress, welfare, anxiolytic, behavior assessments

## Abstract

Nationally, there is a growing population of shelter dogs that are experiencing longer lengths of stay as euthanasia rates decline. Well-documented shelter-specific stressors can induce physiological and behavioral changes correlated with immediate and future welfare concerns. Dogs vary in their responses to the shelter environment based on genetics, past experiences, and context, leaving some individuals unable to adapt. Those that struggle may display maladaptive coping behaviors perceived as less desirable by adopters, perpetuating the stress response cycle. To address welfare concerns, behavioral interventions, including enrichment and training programs, may mitigate stress; however, there remains a population that could benefit from pharmaceutical interventions by supporting individuals at a physiological level. Shelter resources vary significantly, and differences in training, experience, and clinical staff can create challenges to accessing anxiolytic medications. Most shelter behavior assessments focus on temperament testing to predict future behavior at home or identify individuals at risk for aggression, but few exist that can help identify generalized stress related to their current environment. There remains a need for a validated behavior assessment that measures coping ability in a clinically meaningful way to facilitate partnerships between shelters and veterinarians, addressing immediate shelter dog welfare concerns.

## Simple Summary

Pet relinquishment and stray population control present complex societal and animal welfare challenges. Millions of dogs enter shelters annually and with euthanasia rates declining nationally, welfare challenges related to spatial and social restriction are compounded with longer stays. Substantial research on the shelter environment has culminated in a deeper understanding of the physiological and behavioral stress responses experienced by shelter dogs. Those unable to adapt may exhibit maladaptive behaviors, making them less appealing to adopters and prolonging the length of stay or putting individuals at greater risk of euthanasia. To address current welfare needs, several shelters have implemented behavioral or pharmaceutical interventions to mitigate the effects of stress. This review summarizes current research on sheltering, stress mitigation, and extant behavior assessments. Most shelter behavior assessments aim to predict future home behavior without much evidence of success, likely due to the impact of shelter stress on behavioral expression. Few tools assess welfare directly in the animal’s current state, and those that exist mainly identify stress signs without deeper insight into individual needs. Developing behavior assessments focused on coping ability could help staff tailor interventions and allocate resources more effectively, thereby protecting welfare and promoting speedier adoptions.

## 1. Introduction

The management of unowned dog populations represents a persistent and multifaceted challenge for animal welfare and community responsibility. In the United States (US) alone, millions of stray and relinquished dogs enter animal shelters each year. Improved shelter and community efforts to implement best practices, such as specialized medical and behavior care in shelters and increasing partnerships with local private practice veterinarians, has contributed to a decline in euthanasia rates in recent years [1,2,3,4,5]. However, trends also indicate that dogs are experiencing prolonged lengths of stay. Remaining in shelters for longer periods of time leads to intensified exposure to spatial restriction, social deprivation, and environmental unpredictability within shelter settings [6,7,8,9,10,11,12,13,14]. As a social species that evolved alongside humans, removal of a human attachment figure(s) combined with the disparity between the contemporary confines of animal shelters and the capacity to meet the species physical and mental needs, often leaves many dogs struggling to navigate and adapt to their new circumstances [15,16,17,18,19].

A substantial body of research has examined the predictive factors associated with the physiological and behavioral variability of stress in shelter dogs [6,8,9,10,11,12,13,14]. Stress responses are shaped by individual differences, including genetics, early developmental history, prior socialization, and the immediate environmental context. While some dogs demonstrate behavioral flexibility and adapt to shelter conditions, others exhibit maladaptive responses such as heightened stress or fear, repetitive behaviors, withdrawal, or arousal-related behavior problems. Such behaviors may reduce perceived adoptability, thereby extending length of stay and, in some cases, increasing risk for negative outcomes [20,21,22,23,24,25].

In response to these welfare concerns, shelters have implemented a range of environmental, behavioral, and pharmacological interventions aimed at mitigating stress and improving behavioral presentation [26,27,28,29,30,31,32,33,34,35,36]. Enrichment programs, foster placement, training protocols, and anxiolytic therapies have shown varying degrees of success in promoting adaptive coping. Concurrently, numerous behavioral assessment tools have been developed and implemented in shelters with the primary goal of predicting post-adoption behavior and identifying successful matches between dogs and potential adopters. However, evidence supporting the predictive validity of many of these assessments remains limited [37,38]. Behavioral expression in the shelter is influenced by acute and chronic stress, which complicates efforts to extrapolate findings to home environments from those observed in the shelter.

Importantly, most existing assessment frameworks prioritize risk evaluation for aggression rather than the direct measurement of current welfare or coping capacity. A shift toward assessments focused on adaptive functioning in shelters may offer a more welfare-centered approach; by identifying how individual dogs respond to shelter-related challenges, such tools could enable staff to tailor interventions, allocate resources more strategically, and establish faster adoptions to further support canine welfare. While previous reviews have summarized the available literature regarding shelter trends and the sheltering environment, stress mitigation strategies, and behavioral assessment methodologies, this narrative review is framed to highlight the need for collaborative effort between shelter staff, clinicians, and ethologists to develop cost-effective tools to improve canine welfare while housed in an animal shelter. For a list of published reviews that pertain to shelter dog welfare referenced throughout this article, see Appendix A (Table A1).

## 2. Methods

A literary search for published reviews and original research articles was conducted through Google Scholar using the phrases “shelter trends”, “shelter welfare”, “shelter stress”, “shelter behavior assessment”, “temperament test”, “quality of life”, “behavioral modification”, and variations thereof in 2022. Published reviews and articles that pertained to shelter dogs were reviewed for content and included in this review. When lacking shelter dog research, the search was expanded to include clinic, kenneled (working or laboratory), and pet dogs. Relevant textbooks and encyclopedias were referenced when needed to provide background information. Established census reports for the United States sheltering community were accessed to provide context. The original review was included in the author’s thesis publication through ProQuest as partial fulfillment of the requirements for a Master of Science [39]. The literary review underwent further revisions and modifications for the current publication. Prior to publication, the literary search was repeated to include publications that occurred after 2021, and updates were made accordingly. Additional references to novel behavior assessments were included based on the authors’ awareness (i.e., conference proceedings).

## 3. Shelter Trends

Animal shelters provide temporary housing for relinquished pets, strays, and those removed from owners for reasons of neglect or abuse. During this transitional period, dogs are kenneled and cared for in these establishments until they leave under circumstances of return-to-owner, new adoptions, transfers, death by natural causes or euthanasia. The accuracy of shelter dog population estimations at any timepoint is challenged by the lack of a complete national database [3,4,40]. Scholarly estimates for the US shelter population hover around 5 million dogs annually, although estimates from national census data indicate closer to 3.1 million dogs [4,41,42]. In the past, shelter overpopulation, exacerbated by resource shortages, including staff labor, funding, and space, has contributed to high euthanasia rates [20]. Since the 1900s, evolving societal and cultural perceptions on humane animal treatment have led several shelters to enact policies restricting instances of euthanasia to cases of critical illness or poor temperament [2], creating the potential for extended stays, which can further contribute to shelter overpopulation.

Communities have implemented various strategies to address overpopulation, such as encouraging pet retention through supportive programs and moving animals to alternative housing through foster systems. While there has been an effort to identify causes of relinquishment, it remains ill-defined across studies and largely dependent on questionnaires [43,44]. Several reviews emphasize the complexity of relinquishment, with both pet- and owner-related causes at play [26,45,46,47]. More recently, a study focused on dogs and cats in a US open-admission shelter identified 13 categories for relinquishment, with the most common being behavior issues, housing/moving, and the inability to care for the pet [48]. Therefore, relinquishment, in combination with stray intake, continues to fill shelters with available dogs. Data indicates that national euthanasia rates are declining [1,4,5,6] but increasing time spent in-shelter has led researchers to investigate factors that impact length of stay [21,22,23,24,25]. Behavioral factors such as body position in-kennel and activity have been shown to predict longer length of stay in shelters, supporting the belief that behavior influences adopter choice [7,24,25]. Several shelters have implemented obedience and training classes in an attempt to augment the movement of adoptable dogs into homes; however, experimental studies on such programs and the likelihood of adoption show mixed results [26,49,50,51].

Compounded with previously established efforts at decreasing pet overpopulation, the COVID-19 pandemic ushered in a shift in national pet ownership characterized by fewer dogs entering shelters [52]. Additionally, many sources anecdotally reported an increase in adoptions through the onset of the pandemic [53]. Decreased intake and increased live release rates were corroborated by one retrospective study spanning from 2016 to 2020 [1]. However, in contrast, one study found that overall adoption rates have not changed, with evidence of decreased adoptions correlated with a decrease in intake [52]. It is difficult to assess the degree to which the pandemic has influenced shelter trends due to conflicting results across studies, with some showing evidence that shelters were already experiencing decreased intake and increased live release rates [54], while others indicate a pre-pandemic increase in total dog intake [55]. In the years following the onset of the pandemic, census data from 2023 demonstrate a steady increase in intake since 2021 [56].

More recently, the 2025 Shelter Animals Count Annual Data Report shows a 4% decrease in intake compared with 2024, with a 13-day median length of stay, down two days from the previous year [57]. Census data is dependent on organization participation and can fluctuate quarterly, but general trends may indicate that shelters are observing improved occupancy flow, as indicated by shorter lengths of stay. Despite this, dogs that enter shelters continue to experience widely variable lengths of stay, creating situations in which dogs may spend several days to months housed at an animal shelter. On average, census data indicates that shelter dogs are experiencing longer lengths of stay [22,58], resulting in a rising population of shelter dogs awaiting outcomes [59]. To address the needs of those under their care, the shelter community is eager to direct resources toward improving shelter dog welfare, not only to appeal to potential adopters but also to provide individual dogs with the best possible opportunity to successfully transition through the shelter system.

## 4. Environmental Stressors

The effects of shelter-specific environmental and social factors have been studied in dogs using physiological measures of stress such as cortisol, heart rate variability (HRV), and immune function, as well as behavioral measures, including activity level and ethograms, as reviewed by Beerda and colleagues [8], and more recently by Polgar and colleagues [11]. Studies of physiological outcomes in kenneled dogs predominately measure hypothalamic–pituitary–adrenal (HPA) axis activity [12]. The HPA axis is understood to be the primary system activated in response to a stressor. Upon perceiving a threat to homeostasis, the paraventricular nucleus (PVN) of the hypothalamus brain region secretes corticotropin-releasing factor (CRF) to the anterior pituitary gland located near the hypothalamus. The anterior pituitary responds by releasing adreno-corticotropic hormone (ACTH) into circulation, principally targeting the cortex of the adrenal gland. There, glucocorticoids are released into circulation to receptive tissues and organs to prep the body for fight or flight responses. For a review of the HPA axis’s response to stress, see Smith and Vale [60]. Activation of the HPA axis can be measured through levels of glucocorticoids such as cortisol concentration in plasma, saliva, urine, feces, or hair, as reviewed by Polgar and colleagues [11], and more recently by Marza and colleagues [14]. Several environmental and social factors inherent to shelters have thus been identified as stressors that may illicit physiological stress responses and negative affective states in dogs. Shelter-specific stressors, which include spatial and social restriction, loss of attachment figures, sensory overload, and unpredictability due to the novel environment, and their documented effects on dogs were recently reviewed by Protopopova [6].

Kennel environments, as experienced not only by shelter dogs but also working dogs, laboratory animals, veterinary patients, and pets temporarily boarded away from their humans, present environmental stressors inherent to confined housing [6,13]. Constricted activity, high levels of sensory stimuli, and diet contribute to the environmental impact of shelters on dogs. Restricted kennel areas, typically consisting of limited outdoor access and potentially austere conditions, have been shown to increase stress behaviors and cortisol levels [9,61,62]. Limited exercise and barren environments may increase stress by restricting the extent to which a dog can express natural behaviors. In one study, 25 min of human-guided exercise lowered salivary cortisol and improved scores on a behavior test [63]. Sensory stimuli can further contribute to shelter dog stress, as shelter design and management can lead to olfactory and auditory overload. Upon entering a shelter, dogs experience multiple strong odors, including conspecific pheromones and cleaning chemicals, along with persistent exposure to noise from multiple dogs barking, exacerbated in buildings lacking acoustic design [64,65,66]. Ambient shelter noise can reach over 100 dB, which in one study resulted in hearing damage for all dogs exposed for six months [66]. Additionally, diet can have profound effects on physical welfare, often investigated in farm animals [67] and companion animals within the framework of pet nutrition [68]. However, there is only one known study directly investigating the effects of diet on shelter dog cortisol levels in which the authors found that dogs provided with both a premium diet and 20 min of supplemental human interaction per day saw a moderate decrease in plasma cortisol in response to a novel object compared with those provided with human interaction and a maintenance diet or those that received either a maintenance or premium diet with no additional human interaction, suggesting that both human interaction and nutritional enrichment can moderate HPA axis activity [69]. The moderating effects of human interaction found by Hennessy and colleagues [69] has been corroborated by other studies [63,70,71], although the effect is likely context-dependent [12].

Social isolation, both intra-specific and inter-specific, typical of single-housed kennels and constricted time with human contact, respectively, can increase stress [70,72]. In most cases, shelter life comes as an abrupt change from the dog’s previous living arrangement, whether that be in a home or as a stray. Loss of control and predictability, which are characteristics of routine disruption and adherence to novel husbandry practices, cause activation of the HPA axis [12]. This not only impacts the dog physiologically, but when stress becomes chronic, it also induces a negative affective state, leading to poor welfare [13]. Additionally, dogs that enter shelters experience sudden disruption of relationships and separation from attachment figures [73]. Due to the social nature of the species, this too can cause stress, and it has been shown that the presence of a caregiver can reduce canine stress when in a novel environment [73]. Further illustrating the social needs of the species, pair or group housing has been shown to have a positive impact on behavior, and separating bonded dogs can have negative impacts on immune system indicators [72,74]. Studies have found that solitary housing can exacerbate stereotypies [9,74,75], although differences in behavior descriptions complicate the ability to compare across studies, as summarized by Protopopova in Table 1 of her review [6].

While shelter-typical stressors have been identified, staff are limited in their ability to mitigate exposure to these factors. Both available resources and practicality can impede environmental change. Land and building constraints dictate kennel size, sanitation requires the use of cleaning agents, and dogs will bark loudly. Unfortunately, there are several obstacles when addressing shelter dog welfare. For example, additional design consideration requires substantial funding, and increasing social interaction requires available labor to do so. While shelter resources vary, it is unrealistic to assume environmental stressors can be eliminated entirely. To target resources effectively, substantial effort has gone into understanding the effect of environmental factors on the welfare of shelter dogs.

## 5. Coping Ability and Effect on Welfare

Welfare encompasses diverse perspectives on human–animal relationships and pertains to biological, psychological, and natural aspects [76]. Broom, oft referenced, writes “the welfare of an individual is its state as regards its attempt to cope with its environment” [77]. Coping is defined as the behavioral reaction to situations that activate neuroendocrine pathways involved in physiological response to aversive situations [78]. When successful, coping behavior reduces physiological measures of stress and is considered adaptive. However, if the behavior is ineffective at restoring physiological measures to baseline, an individual is at risk for chronic stress and reduced welfare [79].

The shelter environment introduces external stressors documented to activate the HPA axis [12,13]. Prolonged exposure to shelter-typical stressors can lead to dysregulation of the HPA axis, immune suppression, and behavioral changes [6,80]. Beyond the shelter stay, stress experienced in-shelter could have lasting adverse consequences. Psychological stress as experienced by shelter dogs may compromise sociality and cognition, which may increase susceptibility to mental and physical disorders later in life [81] or impact the development of the human–pet bond post-adoption [82]. Therefore, recognizing behavioral indications of maladaptive coping strategies is critical in addressing shelter dog welfare.

Research on stress response supports the adaptive evolution of two different coping styles, proactive or reactive, characterized by consistent behavioral and physiological reactions in response to environmental challenges [78,83,84,85,86]. Animals with a reactive coping style will respond to aversive stimuli with decreased physical activity, commonly demonstrated in animal studies by conditioned immobility, along with increased HPA axis and parasympathetic reactivity [83]. Dogs with a predominately reactive coping style would likely demonstrate anxious-avoidant behaviors and may become despondent or withdrawn. Proactive coping is associated with increased activity, as demonstrated in studies by increased active avoidance, along with decreased HPA axis and parasympathetic reactivity [83]. Dogs that predominately rely on proactive coping strategies may exhibit several behaviors indicative of excessive-arousal and stereotypic activities, such as pacing in-kennel or persistent barking. When animals are placed in situations that fail to fully fit their needs, such as housing systems that restrict natural behavior, individuals may lack innate or learned adaptive coping strategies. For dogs that are incapable of adjusting to the shelter environment, the coping style may impact which behavior patterns manifest while in-shelter.

However, it is important to note that response to stress lies along a continuum of active to inactive physiological and behavioral responses and one individual may rely on a variety of strategies depending on the environmental context, past experiences, and genetics [10,87,88,89]. Extensive research has been done on the behavioral effects of sheltering, with studies generally indicating differences in stereotypical and repetitive behaviors, stress and fear behaviors, and activity level [6]. While relying on behavior alone as an indicator of stress is complicated due to considerable individual variability, species-specific stress signals have been well-documented in kenneled dogs [11,90]. Adoptable dogs generally viewed as less desirable are often those that are struggling to cope with environmental stressors and are exhibiting maladaptive coping behaviors [24,25]. It may take considerably longer for these dogs to get adopted, prolonging exposure to an environment the dog finds stressful.

To encourage successful adoptions, shelters that have the capacity to implement behavioral modification plans to support those that exhibit concerning behavior, typically signals indicative of fear, aggression, or high arousal, have begun to use a holistic approach toward assessment, including context-specific observations (in-kennel, outside, in-building), supplemented with behavioral and/or medical interventions, with the goal of decreasing undesirable behaviors. Dogs that struggle to cope will continue to perform maladaptive behaviors and may be overlooked by potential adopters, ultimately decreasing their welfare due to prolonged stays in the shelter. Behavioral and/or pharmaceutical intervention may help these dogs cope with their environment and ultimately improve their welfare.

## 6. Behavioral Interventions

Behavioral intervention encompasses strategies aimed at mitigating the effects of external stressors. In a review on shelter adoption and relinquishment interventions, Protopopova and Gunter categorize behavioral intervention strategies under object enrichment, sensory enrichment, conspecific interaction, or human interaction [26]. While object enrichment has been shown to influence dog behavior [27,28,29,30], there is no evidence of impact on adoption [27,51]. Sensory enrichment, such as the use of odors and music, as reviewed by Wells [91] and more recently Riggio [92], shows potential to improve welfare, as measured by differences in heart rate variability and behaviors associated with affective state [93,94,95]; however, the mode of exposure confounds the effects of sensory stimuli and complicates conclusions [96]. Conspecific interaction, such as pair-housing, has been a particular area of interest in the sheltering community due to the social nature of the species [97]. Many studies have found that conspecific contact can reduce abnormal behavior, but confounding factors such as pen size and personality impact results [98,99,100]. Other forms of social enrichment include increased positive human interaction [31,32,33,71], such as the behavior-training methods reviewed by Protopopova and Gunter [26], to foster behaviors simultaneously attractive to potential adopters and indicative of improved welfare. In-shelter training may also include habituation and desensitization programs, which aim to decrease sensitivity to specific stimuli that cause individuals’ stress.

Popular support for environmental enrichment to improve welfare has caused a burgeoning collection of scientific literature regarding the benefits and methodology of encouraging species-specific behaviors and maintenance of the healthy physiological reactions essential to homeostasis. While many studies have been conducted on environmental enrichment for kennels, as reviewed by Wells [101] and later by Taylor and Mills [13], sample populations are often restricted by the region served by the shelter, demographic representation of sex, neuter status, age, breed, and disposition, as most participants consist of adoptable dogs deemed medically healthy and available for adoption. Furthermore, as a safety precaution, individuals exhibiting aggressive behavior are often excluded from studies. Therefore, there remains a portion of the shelter dog population that requires additional support for behavior intervention beyond conventional intervention programs.

## 7. Pharmaceutical Interventions

In instances where behavioral intervention programs are ineffective at decreasing concerning behaviors, shelter dogs may benefit from pharmaceutical intervention. Anxiolytic treatment on dogs experiencing anxiety disorder has shown promise [102,103,104]. Anxiolytic medications encompass pharmaceuticals designed to target neural pathways involved in physiological stress responses. However, there have been few studies of therapy drug use specifically on shelter dogs [34,35,36]. This may be largely due to the many obstacles in providing shelter dogs with anxiolytic medications, including the need for a veterinarian to evaluate the dog before prescribing medication. Shelters vary greatly in resources, and not all have veterinarians on staff. Illustrating the disparity in available resources and community need, through a cross-sectional survey of U.S. animal sheltering organizations, Russo and colleagues found that shelter organizations consider lack of access to affordable veterinary and behavioral services a common reason for pet relinquishment. The authors suggested that collaboration between shelters, veterinarians, and animal behaviorists would benefit communities by effectively addressing canine behavior problems [105].

To facilitate collaboration, accurate assessment of individual dogs from shelter staff may save veterinarians time spent on consultations and decrease professional costs. This would alleviate some of the financial burden on shelters to provide dogs with anxiolytic medications. Veterinarian services are partially reliant on third-party observations, and clinical appointments include pet-owner interviews for a better understanding of the animal’s clinical needs. This is necessary, as clinicians are unable to directly observe the animal in all situations. However, communication between veterinarians and caretakers inherently creates potential for a disconnect between actual behavior and perceived meaning. In one study where dog owners, veterinarians, dog trainers, and non-owners were asked to describe dog behavior using adjectives, there was little agreement when classifying behaviors associated with aggression, confidence, and actual play [106]. While this study only involved nine dogs and 60 observers, other studies have shown how experience and education may influence interpretation of animal behavior [107,108]. For example, one study found no difference in ability to interpret behaviors based on self-reported experience factors between 47 veterinary students; however, given that participants were veterinary students, a degree of training and/or familiarity could be assumed [109]. Perhaps more telling, Lilly and colleagues found that shelter volunteers performed poorly on a behavior knowledge survey, although after participating in a six-month training program, scores improved [110]. To ensure quality standard of care, a reliable behavior assessment for shelter staff use would provide veterinarians with accurate information by minimizing observer bias. However, a validated behavior assessment intended to diagnose coping behavior for public use does not currently exist. Instead, behavior assessments used in shelters are focused on temperament testing or broad indication of welfare within shelter establishments.

## 8. Behavior Assessments

Temperament assessments are frequently used in shelters by staff, often to assist in decisions on suitability for adoption. Despite the prevalence of assessments used in shelter management, exact methodology and use varies by shelter [45,111,112]. Evaluating the public safety risk of shelter dogs is an important responsibility of staff and effort has gone into developing assessments that rate temperament to assist in decision-making [113,114,115]. To assess companion pet suitability, temperament tests focus on the dog’s response to certain scenarios designed to mimic potential at-home situations. However, several studies evaluating the predictive validity of temperament testing for shelter dogs have found no correlation between behaviors observed during assessments to post-adoption behavior [114,116,117], with the implication that several dogs are falsely identified as aggressive and deemed unsuitable for adoption [37]. While some studies have utilized principal component analysis to determine the ability of temperament testing to detect personality factors, e.g., fearfulness, friendliness, and aggressiveness, with limited success [118,119], in their review on validation of shelter assessments, Patronek and colleagues conclude that existing temperament assessments used to screen shelter dogs for adoption suitability do not meet accepted standards of validity due to confusion between colloquial and scientific definitions of measurement, limitations in analysis, and the inability to extrapolate beyond the research subjects to all shelter dogs [38]. The discrepancy between in-shelter and post-adoption behavior within an individual can largely be explained by the effects on behavior of the shelter environment and unnatural testing scenarios. Despite the inability to predict behavior, many justify the use of temperament tests as one source of information that can contribute to a broader behavior profile consisting of multiple observations of the dog made in several situations. However, it is important to note that studies have failed to show practical benefits of temperament testing for either the dogs or the adopters. As such, current temperament assessments used in shelters are not suited for evaluating shelter dog welfare.

Beyond temperament tests, there are few shelter-specific quality-of-life (QoL) assessments developed to either assess acclimation to the environment or measure impact of an intervention program, primarily measuring effects of human interaction but also used in some instances for measuring the impacts of varying housing environments. In a review on shelter dog QoL assessments, Lamon and colleagues identified six validated behavior assessments (Table 1) [120]. While these assessments provide a way to compare the welfare of dogs across shelters, results do not indicate individual needs in terms of additional mental support. Furthermore, several are time-consuming and impractical for staff to repeatedly administer to all dogs. Since their review, there has been one project aimed at developing a QoL questionnaire that can be used to assess welfare both in-shelter and at home [121]. However, development is ongoing, and the questionnaire may be better suited to assessing pre-adoption welfare in foster situations. Therefore, there remains a need for a practical behavior assessment that can communicate the current coping state and indicate anxiolytic needs.

**Table 1:** Existing shelter dog QoL assessments. Validation methods for assessments used for evaluating shelter dog welfare published between 2000 and 2020 (adapted from Table 1 in the review by Lamon and colleagues [120]). See [115] for a QoL questionnaire in development.

Assessment Tool	Validation	Study Focus
Quality of Life Assessment (QL)	Quality of life (QL) score calculated using positive and negative indicator behaviors validated against treatment groups	Intervention: Human interaction [122]
		Intervention: Socialization program [123]
Shelter Quality Protocol (SQP)	Management-, resource-, and animal-based measures used to identify welfare hazards; individual measures validated observing individual twice (at a distance and again close-up)	Acclimation [124,125]
Shelter Quality Protocol 2 (SQP 2)	Refined version of SQP validated against climatic conditions	Acclimation [126]
Qualitative Behavioral Assessment (QBA)	Descriptive terms analyzed for consensus dimensions to describe emotional state of shelter dogs	Acclimation [126,127,128] Intervention: Different housing environments and lengths of stay [129]
Multi-operator Qualitative Behavioral Assessment (MQBA)	Stress level score based on brief description of the dog’s behavior; stress level score did not correlate between raters, suggesting the score is situation dependent and not a comprehensive indication of welfare	Acclimation [130]
Approach test	Approach behavior categorized as “contact possible” or “no contact possible” validated against attitude of shelter staff via questionnaire	Intervention: Human interaction [131]
Behavior test	Test is not described in publication; previously validated by authors (not published)	Intervention: Exercise and human contact [63]

Two emerging behavior assessments, the Living and Interactions Functionality Evaluation (LIFE) and the Canine Observed Provision of Enrichment (COPE), aim to quantify shelter dog welfare at the individual level by streamlining communication between shelter staff and veterinarians. The LIFE tool evaluates coping ability by assigning a score based on behaviors observed across routine shelter activities [132]. Recent analysis showed high inter-rater reliability and strong agreement between objective LIFE scores and subjective expert evaluation. Similarly, the COPE tool quantifies coping ability through a behavior assessment developed to mimic common shelter experiences but with the added potential to simultaneously provide enrichment [39,133,134]. A total COPE score with low negative scores indicates maladaptive anxious-avoidant behaviors, high positive scores indicate maladaptive excessive-aroused behaviors, and near zero scores indicate adaptive coping. Scores were validated against diagnoses by a certified veterinarian behaviorist. While both assessments show promise, the tools have not yet been broadly disseminated and will need further analysis across shelters.

## 9. Discussion

Data indicates that dogs are experiencing longer lengths of stay in-shelter, while intake and euthanasia rates decline. Stressors inherent to shelter environments put dogs at risk of behavioral and physiological consequences that can decrease welfare and prolong shelter stay. The ability to cope within the shelter environment is dependent on a variety of factors such as genetics, past experiences, and environmental context. While some dogs can adapt, coping styles may influence the behavioral patterns of those dogs that demonstrate maladaptive coping behaviors. A better understanding of how coping style can impact behavior in-shelter would help clinicians target the appropriate physiological stress response for individualized pharmaceutical interventions.

A significant portion of shelter dogs demonstrate combinations of behaviors indicative of a negative affective state that can be categorized by a coping style. Dogs that are withdrawn, avoidant, and/or despondent show behaviors consistent with a reactive coping style, while dogs that excessively vocalize, are hyper-active, and/or experience loss of impulse control align with what is known about the proactive coping style [83,135]. Current understanding of the physiological processes that influence behavior can guide intervention strategies. There are a substantial number of studies demonstrating the positive effect that enrichment programs can have on shelter dog welfare [92,101]. A smaller but growing body of research is focused on behavior modification programs that encourage behaviors that are appealing to adopters with the goal of decreasing length of stay and increasing adoption rates, although results vary across studies [26]. Beyond enrichment programs and behavioral interventions, pharmaceutical intervention has been shown to decrease anxiety and fear-related phobias in veterinary behavior practice [136,137,138,139]. Shelters with the resources to do so have begun administering anxiolytic medications to support the mental health of dogs exhibiting maladaptive coping behaviors. The few existing shelter-specific studies have found evidence that medication can have a positive effect on dog behavior [34,35,36]; however, there needs to be more research on the efficacy of pharmaceutical treatment for stress management in shelter dogs.

Furthermore, shelter use of anxiolytics is not without controversy. Resistance to medicating shelter dogs often circles around concern of masking problem behaviors and misleading adopters. However, there is no evidence that in-shelter behavior predicts at-home behavior, indicating that, regardless, adopters face a large margin of uncertainty [37,38,44]. Longitudinal studies measuring in-shelter and at-home animal-based parameters may help assuage public doubt. Beyond the public perception, veterinarians vary in their comfort prescribing behavioral medication [140]. A validated assessment tool that would communicate anxiolytic needs would help clinicians make informed decisions and assist in the implementation of individualized interventions within shelters. Current assessments used in shelters are either temperament tests used to determine adoptability or quality of life (QoL) tests used to measure intervention strategies. However, current QoL assessments are unsuited to providing meaningful clinical information. Two novel assessments, the Living and Interactions Functionality Evaluation (LIFE) and the Canine Observed Provision of Enrichment (COPE), were developed to quantify coping behavior, but additional studies on validity, reliability, and practicality for public use are necessary. Future studies on the identification and treatment of dogs unable to cope with the shelter environment should incorporate animal-based measures and pharmaceutical applications to further validate use of the LIFE and COPE tools across shelters.

## 10. Conclusions

Due to the wide variation in dogs’ abilities to adapt to the shelter environment, not all dogs successfully acclimate, resulting in the manifestation of maladaptive coping behaviors that are indicative of a negative affective state and contributory to decreased desirability to potential adopters. When behavioral intervention programs focused on environmental and social enrichment fail to decrease concerning behaviors, anxiolytic pharmaceuticals may benefit the current welfare of shelter dogs. However, resources across shelters varies considerably and not all shelters have access to anxiolytics. Future efforts to develop a reliable and validated assessment for use by shelter staff to quantify coping behavior and communicate meaningful information to veterinarians would facilitate communication between shelters and clinics. The assessment should not only differentiate between dogs in need of medical intervention and those that are adapting but also allow veterinarians to make informed decisions on which medications to prescribe by indicating whether the maladaptive coping behaviors are manifesting as anxious-avoidance or excessive-arousal. By quantifying coping ability, the assessment could also be useful in monitoring efficacy of behavioral interventions through ranked outcomes. Not only would this provide a standardized method to provide individualized care to shelter dogs but also further use of assessment outcomes could result in a national database of shelter dog behavior available for studies on welfare. The emerging LIFE and COPE tools are two such assessments that may provide a validated means to address shelter dog welfare at the individual level.

## Data Availability

No new data were created or analyzed in this study. Data sharing is not applicable to this article.

## Appendix A

**Table A1:** Literature reviews that pertain to shelter dog welfare with focus on stress response, kennel environment, or behavior assessments ^1^.

Category	Topic of Review	Year of Publication	Authors
Stress response	Long-term consequences of shelter stress	2020	Hennessy and colleagues [81]
	Animal-based measures of shelter dog welfare	2019	Polgar and colleagues [11]
	Sheltering effects on physiology and behavior	2016	Protopopova [6]
	Physiological-based measures of shelter dog welfare	2013	Hennessy [12]
	HPA response to stress	2006	Smith and Vale [60]
	Behavioral and neuroendocrinal characteristics of coping styles	1999	Koolhaas and colleagues [83]
Kennel environment	Social enrichment	2019	Walthers and colleagues [97]
	Sensory enrichment	2018	Riggio [92]
	Adoption and relinquishment interventions	2017	Protopopova and Gunter [26]
	Sensory enrichment	2009	Wells [91]
	Physical and psychological stressors	2007	Taylor and Mills [13]
	Environmental enrichment	2004	Wells [101]
	Adoption and relinquishment	2003	Marston and Bennett [45]
Behavior assessments	Existing welfare and quality of life assessment tools	2021	Lamon and colleagues [120]
	Reliability and validity of behavior evaluations	2019	Patronek and colleagues [38]

^1^ This table includes reviews on behavior assessments used in US shelters. Additional reviews have been published regarding behavior assessments used in Australia [141] and the EU [142].
